# Development and suitability of neuropsychological assessment batteries among seniors in Tanzania: Clinical and research implications of the Tanzania 2022 Population and Housing Census

**DOI:** 10.1002/alz70860_097582

**Published:** 2025-12-23

**Authors:** Damas Andrea Mlaki, Victor Valcour, Stella‐Maria Paddick, Isabel Elaine Allen, Andjelika Milicic, Reiner Kiama, Bruce L. Miller

**Affiliations:** ^1^ University of California San Francisco, San Francisco, CA, USA; ^2^ Mirembe National Mental Health Hospital, Dodoma, Dodoma, Tanzania, United Republic of; ^3^ Memory and Aging Center, University of California San Francisco, San Francisco, CA, USA; ^4^ Global Brain Health Institute, University of California, San Francisco, San Francisco, CA, USA; ^5^ Gateshead Health NHS foundation Trust, Department of Old Age Psychiatry, Gateshead, Newcastle Upon Tyne, United Kingdom; ^6^ Department of Epidemiology and Biostatistics, University of California, San Francisco, San Francisco, CA, USA; ^7^ Global Brain Health Institute, University of California, San Francisco, CA, USA, San Francisco, CA, USA, San Francisco, CA, USA; ^8^ Global Brain Health Institute, San Francisco, CA, USA; ^9^ Memory and Aging Center, UCSF Weill Institute forNeurosciences, University of California, San Francisco, San Francisco, CA, USA, San Francisco, CA, USA; ^10^ National Bureau of Statistics, Dodoma, Dodoma, Tanzania, United Republic of; ^11^ Memory and Aging Center, Weill Institute for Neurosciences, University of California, San Francisco, San Francisco, CA, USA; ^12^ Department of Neurology, Memory and Aging Center, University of California San Francisco, San Francisco, CA, USA

## Abstract

**Background:**

Currently, dementia affects over 57 million people across the world and over two‐thirds live in low‐and‐middle‐income countries (LMICs) where over 75% (28.5million) remains undiagnosed. The identification of dementia using valid and reliable measures of cognitive impairment is crucial for the development of effective preventive interventions, treatments and care plans. We analysed the 2022 Population and Housing Census data in Tanzania to determine the age‐adjusted prevalence of subjective memory, hearing and visual complaints and the factors that may influence development and suitability of neuropsychological assessment batteries among seniors. In addition, we explored the pathways by which age, education and gender influence subjective memory complaints

**Method:**

We extracted data for the older adults aged 60+ on literacy, numeracy, hearing, visual and memory complaints. Age‐adjusted prevalence estimates were calculated using the WHO Direct Method. Logistic regression models were performed to examine the factors associated with subjective memory complaints. Mediation analysis was conducted using path analysis and significance of the indirect effects was tested using bootstrapping procedures.

**Result:**

Adults aged 60+ constituted 5.7% (3,491,983) of the population. The median (IQR) age was 60 (60–97) years. Literacy and numeracy rates were 59.7 % and 67.7 % respectively and decreased with age. The age‐ adjusted prevalence of subjective memory, hearing and visual complaints, were 7.3% (95%CI: 7.2–7.4), 7.5% (95%CI: 7.4–7.6) and 16.3% (95%CI: 16.2– 16.4). Being married or living together (AOR 0.83; 95%CI: 0.74–0.93; *p* = 0.002) and having a 7‐year education level and over (*p* ≤0.001) reduced the odds of memory complaints, while hearing (AOR=4.62; 95%CI: 4.37–4.88; *p* ≤0.001) and visual (AOR 7.12; 95%CI: 6.78–7.47; *p* ≤0.001) complaints increased the likelihood of memory complaints. Age (*p* ≤0.001) and female gender (*p* ≤0.001) accounted for 21% and 7% of the effects of gender and education on subjective memory complaints respectively.

**Conclusion:**

Literacy and numeracy decrease with increasing age in those over 60. Hearing and visual complaints are predominant among seniors and increase the risk of subjective memory complaints. Our findings may inform the development and suitability of neuropsychological assessment batteries among seniors in Tanzania.

